# 6-Chloro-8-nitro-4-oxo-4*H*-chromene-3-carbaldehyde

**DOI:** 10.1107/S1600536814007788

**Published:** 2014-04-12

**Authors:** Yoshinobu Ishikawa

**Affiliations:** a School of Pharmaceutical Sciences, University of Shizuoka, 52-1 Yada, Suruga-ku, Shizuoka 422-8526, Japan

## Abstract

In the title compound, C_10_H_4_ClNO_5_, the non-H atoms of the 6-chloro­chromone unit are coplanar (r.m.s. deviation = 0.017 Å) with the largest deviation from the mean plane [0.031 (2) Å] being found for the C=O C atom. The nitro group (NO_2_) is inclined to the chromone unit mean plane by 13.3 (2) °. The formyl group is also twisted with respect to the attached ring [C—C—C—O torsion angles = 10.8 (4) and −171.8 (2)°]. In the crystal, mol­ecules are linked via C-H⋯O hydrogen bonds forming slab-like networks lying parallel to (-301). The slabs are linked by π–π inter­actions involving the benzene rings of the chromone units [centroid–centroid distance = 3.770 (3) Å].

## Related literature   

For related structures, see: Ishikawa & Motohashi (2013 ▶); Ishikawa (2014 ▶). For halogen bonding, see: Auffinger *et al.* (2004 ▶); Metrangolo *et al.* (2005 ▶); Wilcken *et al.* (2013 ▶); Sirimulla *et al.* (2013 ▶).
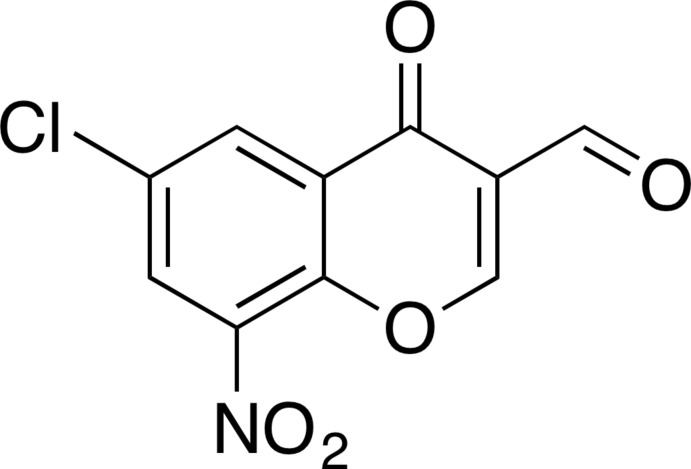


## Experimental   

### 

#### Crystal data  

C_10_H_4_ClNO_5_
*M* *_r_* = 253.60
Monoclinic, 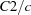

*a* = 18.585 (9) Å
*b* = 10.4918 (17) Å
*c* = 11.094 (3) Å
β = 119.23 (3)°
*V* = 1887.7 (12) Å^3^
*Z* = 8
Mo *K*α radiation
μ = 0.41 mm^−1^
*T* = 100 K
0.38 × 0.22 × 0.18 mm

#### Data collection  

Rigaku AFC-7R diffractometer
2588 measured reflections
2173 independent reflections
1903 reflections with *F* ^2^ > 2σ(*F* ^2^)
*R*_int_ = 0.019
3 standard reflections every 150 reflections intensity decay: −0.7%

#### Refinement  

*R*[*F* ^2^ > 2σ(*F* ^2^)] = 0.035
*wR*(*F* ^2^) = 0.098
*S* = 1.05
2173 reflections
154 parameters
H-atom parameters constrained
Δρ_max_ = 0.29 e Å^−3^
Δρ_min_ = −0.38 e Å^−3^

### 

Data collection: *WinAFC Diffractometer Control Software* (Rigaku, 1999 ▶); cell refinement: *WinAFC Diffractometer Control Software*; data reduction: *WinAFC Diffractometer Control Software*; program(s) used to solve structure: *SIR2008* (Burla *et al.*, 1989 ▶); program(s) used to refine structure: *SHELXL97* (Sheldrick, 2008 ▶); molecular graphics: *CrystalStructure* (Rigaku, 2010 ▶); software used to prepare material for publication: *CrystalStructure*.

## Supplementary Material

Crystal structure: contains datablock(s) General, I. DOI: 10.1107/S1600536814007788/tk5305sup1.cif

Structure factors: contains datablock(s) I. DOI: 10.1107/S1600536814007788/tk5305Isup2.hkl

Supporting information file. DOI: 10.1107/S1600536814007788/tk5305Isup3.cml

CCDC reference: 996005

Additional supporting information:  crystallographic information; 3D view; checkCIF report

## Figures and Tables

**Table 1:** Hydrogen-bond geometry (Å, °)

*D*—H⋯*A*	*D*—H	H⋯*A*	*D*⋯*A*	*D*—H⋯*A*
C1—H1⋯O4^i^	0.95	2.53	3.463 (3)	169
C4—H2⋯O2^ii^	0.95	2.35	3.250 (3)	158
C6—H3⋯O5^iii^	0.95	2.27	3.191 (3)	164

Symmetry codes: (i) 
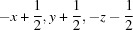
; (ii) 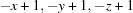; (iii) 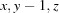.

**Fig. 1.**
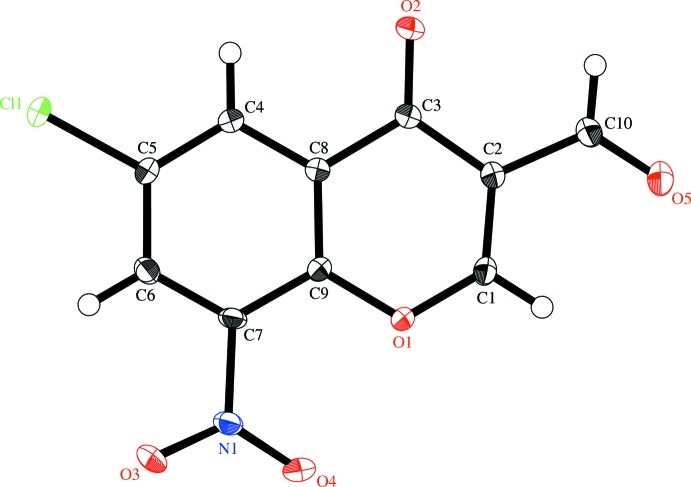
The molecular structure of the title compound with displacement ellipsoids drawn at the 50% probability level. Hydrogen atoms are shown as small spheres of arbitrary radius.

**Fig. 2.**
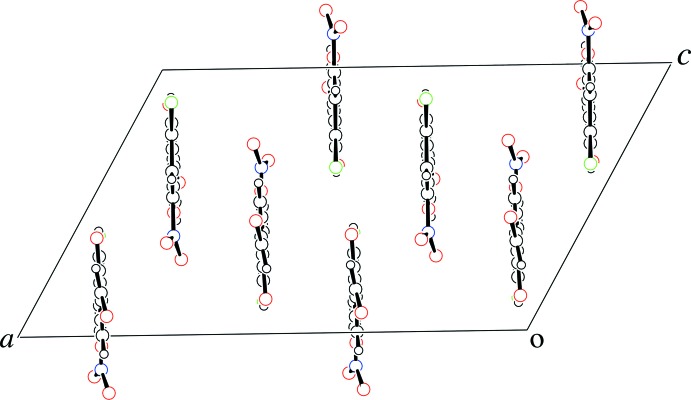
A packing view of the title compound.

**Fig. 3.**
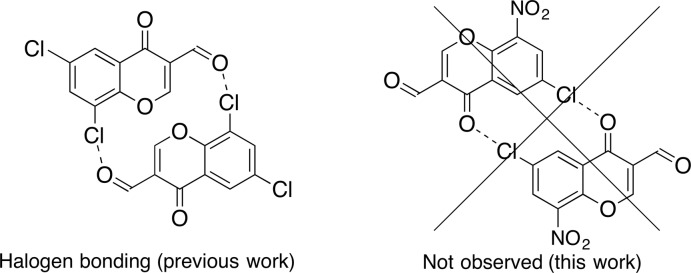
An illustration of the structure of 6,8-dichloro-4-oxochromene-3-carbaldehyde with halogen bonds in the crystal (left) and a hypothetical model of the title compound with halogen bonds (right).

## supplementary crystallographic information

### 1. Structural commentary

Halogen bonds have been found to occur in organic, inorganic, and biological systems, and have recently attracted much attention in medicinal chemistry, chemical biology and supra­molecular chemistry (Auffinger *et al.*, 2004; Metrangolo *et al.*, 2005; Wilcken *et al.*, 2013; Sirimulla *et al.*, 2013). We have recently reported the crystal structures of a dichlorinated 3-formyl­chromone derivative 6,8-di­chloro-4-oxochromene-3-carbaldehyde (Ishikawa & Motohashi, 2013) and a monochlorinated 3-formyl­chromone derivative 6-chloro-4-oxo-4*H*-chromene-3-carbaldehyde (Ishikawa, 2014). It was found that halogen bonding is observed between the formyl oxygen atom and the chlorine atom at 8-position (Fig. 1, left), and is not observed between any oxygen atom and the chlorine atom at 6-position. As part of our inter­est in this type of chemical bonding, we herein report the crystal structure of a monochlorinated 3-formyl­chromone derivative with a nitro group, 6-chloro-8-nitro-4-oxo-4*H*-chromene-3-carbaldehyde. The objective of this study is to reveal whether halogen bond(*s*) can be formed in the crystal structure of this compound with an electron-withdrawing group near the chlorine atom at 6-position. It was postulated that the size of a σ hole of the chlorine atom might be large enough to form halogen bond(*s*) by electron-withdrawing inductive effect of the nitro group (Wilcken *et al.*, 2013).

The mean deviation of the least-square planes for the non-hydrogen atoms of the 6-chloro­chromone unit is 0.0228 Å, and the largest deviations is 0.054 (2) Å for C2 (Fig. 2). The nitro group is twisted from this plane as seen in the dihedral angle between the least-squares planes of 14.116 (10) Å. The formyl group is also twisted [C1–C2–C10–O5 = 10.8 (4)° and C3–C2–C10–O5 = -171.8 (2)°], as shown in Fig. 2.

In the crystal, the molecules are linked through stacking inter­action along the *a* axis [centroid–centroid distance between the benzene rings of the chromone units = 3.770 (3) Å], as shown in Fig. 3. The distances between the chlorine atom and the oxygen atoms of the nitro group [3.874 (2) Å], the formyl group [3.535 (3) and 3.666 (2) Å], and the α,β-unsaturated carbonyl group [3.595 (2) Å] are far from halogen bonding. A structure with halogen bonds can be envisaged for the title compound (Fig. 1, right), but it is not observed in the present crystal structure.

### 2. Synthesis and crystallization

To a solution of 5'-chloro-2'-hy­droxy-3'-nitro­aceto­phenone (3.5 mmol) in *N*,*N*-di­methyl­formamide (10 ml) was added dropwise POCl_3_ (8.7 mmol) for 5 min at 0 °C. After the mixture was stirred for 14 h at room temperature, water (40 ml) was added. The precipitates were collected, washed with water and dried *in vacuo.* Recrystallization from ethyl acetate gave yellow crystals (yield: 25%). ^1^H NMR (400 MHz, DMSO-*d*_6_): *δ* = 8.40 (d, 1H, *J* = 2.4 Hz), 8.71 (d, 1H, *J* = 2.4 Hz), 9.06 (s, 1H), 10.08 (s, 1H). DART-MS calcd for [C_10_H_4_Cl_1_N_1_O_5_ + H^+^]: 253.978, found 254.005.

### 3. Refinement

The C(*sp*^2^)-bound hydrogen atoms were placed in geometrical positions [C–H 0.95 Å, *U*_iso_(H) = 1.2*U*_eq_(C)], and refined using a riding model.

### Crystal data

**Table:**

C_10_H_4_ClNO_5_	*F*(000) = 1024.00
*M**_r_* = 253.60	*D*_x_ = 1.785 Mg m^−^^3^
Monoclinic, *C*2/*c*	Mo *K*α radiation, λ = 0.71069 Å
Hall symbol: -C 2yc	Cell parameters from 25 reflections
*a* = 18.585 (9) Å	θ = 15.4–17.5°
*b* = 10.4918 (17) Å	µ = 0.41 mm^−^^1^
*c* = 11.094 (3) Å	*T* = 100 K
β = 119.23 (3)°	Plate, yellow
*V* = 1887.7 (12) Å^3^	0.38 × 0.22 × 0.18 mm
*Z* = 8	

### Data collection

**Table:**

Rigaku AFC-7R diffractometer	θ_max_ = 27.5°
ω–2θ scans	*h* = −13→24
2588 measured reflections	*k* = 0→13
2173 independent reflections	*l* = −14→12
1903 reflections with *F*^2^ > 2σ(*F*^2^)	3 standard reflections every 150 reflections
*R*_int_ = 0.019	intensity decay: −0.7%

### Refinement

**Table:**

Refinement on *F*^2^	Secondary atom site location: difference Fourier map
*R*[*F*^2^ > 2σ(*F*^2^)] = 0.035	Hydrogen site location: inferred from neighbouring sites
*wR*(*F*^2^) = 0.098	H-atom parameters constrained
*S* = 1.05	*w* = 1/[σ^2^(*F*_o_^2^) + (0.0464*P*)^2^ + 3.8081*P*] where *P* = (*F*_o_^2^ + 2*F*_c_^2^)/3
2173 reflections	(Δ/σ)_max_ < 0.001
154 parameters	Δρ_max_ = 0.29 e Å^−^^3^
0 restraints	Δρ_min_ = −0.38 e Å^−^^3^
Primary atom site location: structure-invariant direct methods	

### Special details

**Table:**

Refinement. Refinement was performed using all reflections. The weighted *R*-factor (*wR*) and goodness of fit (*S*) are based on *F*^2^. *R*-factor (gt) are based on *F*. The threshold expression of *F*^2^ > 2.0 σ(*F*^2^) is used only for calculating *R*-factor (gt).

### Fractional atomic coordinates and isotropic or equivalent isotropic displacement parameters (Å^2^)

**Table:**

	*x*	*y*	*z*	*U*_iso_*/*U*_eq_	
Cl1	0.44735 (3)	0.12307 (4)	0.37638 (4)	0.02112 (14)	
O1	0.32475 (8)	0.53278 (12)	−0.03825 (12)	0.0162 (3)	
O2	0.44970 (8)	0.63260 (12)	0.36926 (13)	0.0197 (3)	
O3	0.30625 (11)	0.16061 (14)	−0.15276 (15)	0.0321 (4)	
O4	0.25980 (8)	0.35147 (13)	−0.21525 (13)	0.0220 (3)	
O5	0.34664 (10)	0.91339 (13)	0.07444 (15)	0.0259 (4)	
N1	0.30008 (9)	0.27227 (15)	−0.12702 (15)	0.0172 (3)	
C1	0.33433 (11)	0.65518 (17)	0.00207 (18)	0.0164 (4)	
C2	0.37408 (11)	0.69511 (16)	0.13480 (18)	0.0151 (4)	
C3	0.41012 (11)	0.60278 (16)	0.24645 (17)	0.0146 (4)	
C4	0.42517 (10)	0.37048 (16)	0.29954 (17)	0.0143 (4)	
C5	0.41278 (10)	0.24532 (16)	0.25645 (17)	0.0152 (4)	
C6	0.37215 (10)	0.21418 (17)	0.11583 (18)	0.0157 (4)	
C7	0.34310 (10)	0.31087 (17)	0.01960 (17)	0.0144 (4)	
C8	0.39566 (10)	0.46805 (16)	0.20106 (17)	0.0132 (4)	
C9	0.35390 (10)	0.43954 (16)	0.06022 (17)	0.0136 (4)	
C10	0.38301 (12)	0.83413 (18)	0.16297 (19)	0.0191 (4)	
H1	0.3114	0.7185	−0.0679	0.0197*	
H2	0.4535	0.3903	0.3953	0.0172*	
H3	0.3647	0.1276	0.0872	0.0188*	
H4	0.4189	0.8622	0.2546	0.0229*	

### Atomic displacement parameters (Å^2^)

**Table:**

	*U*^11^	*U*^22^	*U*^33^	*U*^12^	*U*^13^	*U*^23^
Cl1	0.0291 (3)	0.0136 (3)	0.0160 (3)	0.00127 (17)	0.00746 (18)	0.00454 (15)
O1	0.0234 (7)	0.0120 (6)	0.0106 (6)	0.0016 (5)	0.0061 (5)	0.0011 (5)
O2	0.0231 (7)	0.0158 (7)	0.0127 (6)	−0.0004 (5)	0.0029 (6)	−0.0029 (5)
O3	0.0562 (11)	0.0153 (7)	0.0186 (7)	−0.0017 (7)	0.0133 (7)	−0.0070 (6)
O4	0.0250 (7)	0.0227 (7)	0.0114 (6)	0.0001 (6)	0.0035 (6)	−0.0006 (5)
O5	0.0394 (9)	0.0143 (7)	0.0239 (7)	0.0011 (6)	0.0154 (7)	0.0019 (6)
N1	0.0220 (8)	0.0170 (8)	0.0125 (7)	−0.0043 (6)	0.0083 (6)	−0.0037 (6)
C1	0.0212 (9)	0.0119 (8)	0.0159 (9)	0.0026 (7)	0.0089 (7)	0.0024 (7)
C2	0.0179 (8)	0.0120 (8)	0.0148 (8)	0.0005 (7)	0.0075 (7)	−0.0002 (7)
C3	0.0158 (8)	0.0137 (8)	0.0128 (8)	−0.0005 (7)	0.0059 (7)	−0.0011 (7)
C4	0.0156 (8)	0.0140 (9)	0.0114 (8)	0.0000 (7)	0.0050 (7)	0.0004 (7)
C5	0.0173 (8)	0.0134 (8)	0.0133 (8)	0.0016 (7)	0.0063 (7)	0.0028 (7)
C6	0.0190 (8)	0.0127 (8)	0.0162 (8)	−0.0022 (7)	0.0092 (7)	−0.0024 (7)
C7	0.0167 (8)	0.0158 (8)	0.0100 (8)	−0.0027 (7)	0.0059 (7)	−0.0035 (7)
C8	0.0146 (8)	0.0125 (8)	0.0118 (8)	−0.0003 (6)	0.0059 (7)	−0.0005 (6)
C9	0.0158 (8)	0.0128 (8)	0.0116 (8)	0.0010 (6)	0.0062 (7)	0.0013 (6)
C10	0.0250 (10)	0.0136 (9)	0.0187 (9)	−0.0025 (7)	0.0107 (8)	−0.0013 (7)

### Geometric parameters (Å, º)

**Table:**

Cl1—C5	1.7301 (18)	C3—C8	1.480 (3)
O1—C1	1.343 (3)	C4—C5	1.378 (3)
O1—C9	1.366 (2)	C4—C8	1.399 (3)
O2—C3	1.231 (2)	C5—C6	1.400 (3)
O3—N1	1.224 (3)	C6—C7	1.377 (3)
O4—N1	1.2220 (19)	C7—C9	1.406 (3)
O5—C10	1.210 (3)	C8—C9	1.396 (3)
N1—C7	1.476 (3)	C1—H1	0.950
C1—C2	1.352 (3)	C4—H2	0.950
C2—C3	1.453 (3)	C6—H3	0.950
C2—C10	1.484 (3)	C10—H4	0.950
			
O1···O4	2.5723 (18)	C6···N1^iii^	3.266 (4)
O1···N1	2.865 (2)	C6···C5^v^	3.544 (4)
O1···C3	2.853 (3)	C6···C7^iii^	3.530 (3)
O2···C1	3.566 (3)	C7···O2^vi^	3.207 (3)
O2···C4	2.833 (3)	C7···N1^iii^	3.516 (4)
O2···C10	2.911 (3)	C7···C6^iii^	3.530 (3)
O3···C6	2.670 (3)	C7···C7^iii^	3.511 (4)
O3···C9	3.590 (3)	C8···C4^v^	3.487 (4)
O4···C6	3.526 (3)	C8···C8^v^	3.479 (4)
O4···C9	2.834 (3)	C9···O2^vi^	3.452 (3)
O5···C1	2.803 (3)	C10···O3^iv^	3.011 (4)
C1···C8	2.751 (3)	C10···N1^iv^	3.544 (4)
C2···C9	2.777 (3)	C10···C1^viii^	3.529 (4)
C4···C7	2.781 (3)	Cl1···H2	2.8102
C5···C9	2.786 (3)	Cl1···H3	2.8003
C6···C8	2.789 (3)	O2···H2	2.5550
Cl1···Cl1^i^	3.5748 (10)	O2···H4	2.6525
Cl1···O2^ii^	3.5947 (15)	O3···H3	2.3584
Cl1···O4^iii^	3.371 (3)	O5···H1	2.4692
Cl1···O5^iv^	3.535 (3)	N1···H3	2.5702
Cl1···C6^v^	3.448 (3)	C1···H4	3.2764
O1···O2^vi^	3.433 (3)	C3···H1	3.2794
O1···O3^vii^	3.365 (2)	C3···H2	2.6539
O1···O5^viii^	3.062 (3)	C3···H4	2.7252
O1···C4^vi^	3.321 (3)	C4···H3	3.2742
O2···Cl1^ii^	3.5947 (15)	C6···H2	3.2764
O2···O1^iv^	3.433 (3)	C9···H1	3.1800
O2···O3^iv^	3.352 (3)	C9···H2	3.2847
O2···O4^iv^	3.183 (3)	C9···H3	3.2837
O2···N1^iv^	2.974 (3)	C10···H1	2.5450
O2···C1^v^	3.551 (3)	H1···H4	3.4766
O2···C2^v^	3.362 (4)	Cl1···H1^iv^	3.3241
O2···C3^v^	3.435 (4)	Cl1···H3^v^	3.3146
O2···C4^ii^	3.250 (3)	Cl1···H4^x^	2.9832
O2···C7^iv^	3.207 (3)	O1···H2^vi^	2.9400
O2···C9^iv^	3.452 (3)	O2···H2^ii^	2.3513
O3···O1^ix^	3.365 (2)	O3···H1^ix^	2.8519
O3···O2^vi^	3.352 (3)	O3···H4^vi^	2.7560
O3···O4^ix^	3.524 (2)	O4···H1^ix^	2.5249
O3···O5^x^	3.435 (3)	O4···H3^iii^	3.2607
O3···O5^vi^	3.542 (3)	O5···H1^viii^	3.2161
O3···C1^ix^	3.458 (3)	O5···H3^xi^	2.2666
O3···C2^vi^	3.512 (4)	N1···H1^ix^	3.0357
O3···C10^vi^	3.011 (4)	N1···H3^iii^	3.4641
O4···Cl1^iii^	3.371 (3)	N1···H4^vi^	3.3795
O4···O2^vi^	3.183 (3)	C1···H1^viii^	3.4007
O4···O3^vii^	3.524 (2)	C1···H2^vi^	3.0167
O4···O5^ix^	3.538 (3)	C2···H1^viii^	3.2806
O4···C1^ix^	3.463 (3)	C3···H2^ii^	3.5197
O4···C2^vi^	3.312 (4)	C4···H1^iv^	3.2457
O4···C3^vi^	3.062 (4)	C5···H1^iv^	3.3272
O4···C5^iii^	3.177 (3)	C7···H2^v^	3.5199
O4···C6^iii^	3.217 (3)	C7···H3^iii^	3.5024
O5···Cl1^vi^	3.535 (3)	C8···H2^v^	3.5485
O5···O1^viii^	3.062 (3)	C9···H2^v^	3.4053
O5···O3^xi^	3.435 (3)	C10···H1^viii^	3.2794
O5···O3^iv^	3.542 (3)	C10···H3^xi^	3.1660
O5···O4^vii^	3.538 (3)	C10···H4^v^	3.3452
O5···C1^viii^	3.124 (4)	H1···Cl1^vi^	3.3241
O5···C6^xi^	3.191 (3)	H1···O3^vii^	2.8519
N1···O2^vi^	2.974 (3)	H1···O4^vii^	2.5249
N1···C2^vi^	3.541 (4)	H1···O5^viii^	3.2161
N1···C3^vi^	3.267 (4)	H1···N1^vii^	3.0357
N1···C5^iii^	3.492 (3)	H1···C1^viii^	3.4007
N1···C6^iii^	3.266 (4)	H1···C2^viii^	3.2806
N1···C7^iii^	3.516 (4)	H1···C4^vi^	3.2457
N1···C10^vi^	3.544 (4)	H1···C5^vi^	3.3272
C1···O2^v^	3.551 (3)	H1···C10^viii^	3.2794
C1···O3^vii^	3.458 (3)	H1···H1^viii^	3.3600
C1···O4^vii^	3.463 (3)	H1···H2^vi^	3.0810
C1···O5^viii^	3.124 (4)	H2···O1^iv^	2.9400
C1···C4^vi^	3.417 (4)	H2···O2^ii^	2.3513
C1···C10^viii^	3.529 (4)	H2···C1^iv^	3.0167
C2···O2^v^	3.362 (4)	H2···C3^ii^	3.5197
C2···O3^iv^	3.512 (4)	H2···C7^v^	3.5199
C2···O4^iv^	3.312 (4)	H2···C8^v^	3.5485
C2···N1^iv^	3.541 (4)	H2···C9^v^	3.4053
C3···O2^v^	3.435 (4)	H2···H1^iv^	3.0810
C3···O4^iv^	3.062 (4)	H2···H2^ii^	3.1238
C3···N1^iv^	3.267 (4)	H3···Cl1^v^	3.3146
C3···C3^v^	3.303 (4)	H3···O4^iii^	3.2607
C4···O1^iv^	3.321 (3)	H3···O5^x^	2.2666
C4···O2^ii^	3.250 (3)	H3···N1^iii^	3.4641
C4···C1^iv^	3.417 (4)	H3···C7^iii^	3.5024
C4···C4^v^	3.454 (4)	H3···C10^x^	3.1660
C4···C8^v^	3.487 (4)	H3···H4^x^	3.2237
C5···O4^iii^	3.177 (3)	H4···Cl1^xi^	2.9832
C5···N1^iii^	3.492 (3)	H4···O3^iv^	2.7560
C5···C5^v^	3.314 (4)	H4···N1^iv^	3.3795
C5···C6^v^	3.544 (4)	H4···C10^v^	3.3452
C6···Cl1^v^	3.448 (3)	H4···H3^xi^	3.2237
C6···O4^iii^	3.217 (3)	H4···H4^v^	3.0665
C6···O5^x^	3.191 (3)		
			
C1—O1—C9	118.83 (14)	N1—C7—C9	122.16 (15)
O3—N1—O4	123.66 (15)	C6—C7—C9	121.20 (16)
O3—N1—C7	117.23 (14)	C3—C8—C4	119.75 (15)
O4—N1—C7	119.10 (16)	C3—C8—C9	119.65 (15)
O1—C1—C2	124.91 (16)	C4—C8—C9	120.59 (16)
C1—C2—C3	120.08 (16)	O1—C9—C7	119.50 (15)
C1—C2—C10	118.65 (16)	O1—C9—C8	121.89 (15)
C3—C2—C10	121.21 (15)	C7—C9—C8	118.60 (15)
O2—C3—C2	123.47 (16)	O5—C10—C2	122.94 (16)
O2—C3—C8	122.01 (15)	O1—C1—H1	117.543
C2—C3—C8	114.53 (14)	C2—C1—H1	117.546
C5—C4—C8	119.41 (16)	C5—C4—H2	120.299
Cl1—C5—C4	120.22 (13)	C8—C4—H2	120.290
Cl1—C5—C6	118.66 (14)	C5—C6—H3	120.469
C4—C5—C6	121.12 (16)	C7—C6—H3	120.471
C5—C6—C7	119.06 (17)	O5—C10—H4	118.529
N1—C7—C6	116.64 (16)	C2—C10—H4	118.529
			
C1—O1—C9—C7	178.93 (17)	C5—C4—C8—C3	−178.83 (17)
C1—O1—C9—C8	−2.2 (3)	C5—C4—C8—C9	0.1 (3)
C9—O1—C1—C2	2.8 (3)	C8—C4—C5—Cl1	−179.02 (16)
C9—O1—C1—H1	−177.2	C8—C4—C5—C6	0.9 (3)
O3—N1—C7—C6	−12.8 (3)	H2—C4—C5—Cl1	1.0
O3—N1—C7—C9	167.45 (19)	H2—C4—C5—C6	−179.1
O4—N1—C7—C6	166.20 (17)	H2—C4—C8—C3	1.2
O4—N1—C7—C9	−13.5 (3)	H2—C4—C8—C9	−179.9
O1—C1—C2—C3	−0.2 (4)	Cl1—C5—C6—C7	178.84 (13)
O1—C1—C2—C10	177.18 (18)	Cl1—C5—C6—H3	−1.1
H1—C1—C2—C3	179.8	C4—C5—C6—C7	−1.0 (3)
H1—C1—C2—C10	−2.8	C4—C5—C6—H3	179.0
C1—C2—C3—O2	177.3 (2)	C5—C6—C7—N1	−179.42 (17)
C1—C2—C3—C8	−2.7 (3)	C5—C6—C7—C9	0.3 (3)
C1—C2—C10—O5	10.8 (4)	H3—C6—C7—N1	0.6
C1—C2—C10—H4	−169.2	H3—C6—C7—C9	−179.7
C3—C2—C10—O5	−171.8 (2)	N1—C7—C9—O1	−0.8 (3)
C3—C2—C10—H4	8.2	N1—C7—C9—C8	−179.69 (17)
C10—C2—C3—O2	−0.1 (4)	C6—C7—C9—O1	179.53 (18)
C10—C2—C3—C8	−179.99 (19)	C6—C7—C9—C8	0.6 (3)
O2—C3—C8—C4	2.1 (4)	C3—C8—C9—O1	−0.8 (3)
O2—C3—C8—C9	−176.81 (19)	C3—C8—C9—C7	178.10 (17)
C2—C3—C8—C4	−177.99 (18)	C4—C8—C9—O1	−179.68 (18)
C2—C3—C8—C9	3.1 (3)	C4—C8—C9—C7	−0.8 (3)

Symmetry codes: (i) −*x*+1, −*y*, −*z*+1; (ii) −*x*+1, −*y*+1, −*z*+1; (iii) −*x*+1/2, −*y*+1/2, −*z*; (iv) *x*, −*y*+1, *z*+1/2; (v) −*x*+1, *y*, −*z*+1/2; (vi) *x*, −*y*+1, *z*−1/2; (vii) −*x*+1/2, *y*+1/2, −*z*−1/2; (viii) −*x*+1/2, −*y*+3/2, −*z*; (ix) −*x*+1/2, *y*−1/2, −*z*−1/2; (x) *x*, *y*−1, *z*; (xi) *x*, *y*+1, *z*.

### Hydrogen-bond geometry (Å, º)

**Table:**

*D*—H···*A*	*D*—H	H···*A*	*D*···*A*	*D*—H···*A*
C1—H1···O4^vii^	0.95	2.53	3.463 (3)	169
C4—H2···O2^ii^	0.95	2.35	3.250 (3)	158
C6—H3···O5^x^	0.95	2.27	3.191 (3)	164

Symmetry codes: (ii) −*x*+1, −*y*+1, −*z*+1; (vii) −*x*+1/2, *y*+1/2, −*z*−1/2; (x) *x*, *y*−1, *z*.
